# Strategies for Enhancing Polyester-Based Materials for Bone Fixation Applications

**DOI:** 10.3390/molecules26040992

**Published:** 2021-02-13

**Authors:** Raasti Naseem, Charalampos Tzivelekis, Matthew J. German, Piergiorgio Gentile, Ana M. Ferreira, Kenny Dalgarno

**Affiliations:** 1 School of Engineering, Newcastle University, Newcastle upon Tyne NE1 7RU, UK; piergiorgio.gentile@newcastle.ac.uk (P.G.); ana.ferreira-duarte@newcastle.ac.uk (A.M.F.); kenny.dalgarno@newcastle.ac.uk (K.D.); 2 School of Dental Sciences, Translational and Clinical Research Institute, Faculty of Medical Sciences, Newcastle University, Newcastle upon Tyne NE1 7RU, UK; babis.tzivelekis@newcastle.ac.uk (C.T.); matthew.german@newcastle.ac.uk (M.J.G.)

**Keywords:** biomaterials, polyesters, polymer blends, copolymers, biodegradable materials, bone regeneration, mechanical properties, composites, glass fibres

## Abstract

Polyester-based materials are established options, regarding the manufacturing of bone fixation devices and devices in routine clinical use. This paper reviews the approaches researchers have taken to develop these materials to improve their mechanical and biological performances. Polymer blending, copolymerisation, and the use of particulates and fibre bioceramic materials to make composite materials and surface modifications have all been studied. Polymer blending, copolymerisation, and particulate composite approaches have been adopted commercially, with the primary focus on influencing the in vivo degradation rate. There are emerging opportunities in novel polymer blends and nanoscale particulate systems, to tune bulk properties, and, in terms of surface functionalisation, to optimise the initial interaction of devices with the implanted environment, offering the potential to improve the clinical performances of fracture fixation devices.

## 1. Introduction

Fractured bones require stabilisation to allow healing to occur. For complex fractures, and in anatomical areas not conducive to external fixation, internal fracture fixation plates are required. Currently, titanium alloys are the most commonly used materials for these plates due to their strength and stiffness being high enough to protect the damaged bone during healing. However, once healing is complete, these high mechanical properties can become problematic, in particular, disrupting the normal remodelling processes of the bone, leading to a reduction in bone quality under the plates, termed stress shielding [1,2]. In fact, stress shielding can become so sufficiently severe that a significant number of revision operations are required annually to remove the fracture plates—along with the associated costs to health care funders and health risks for patients [3].

To reduce the incidence of post-operative stress-shielding research has focused on developing alternative materials for use in fracture plates. In particular, attractive materials are polymers that dissolve, or resorb, in aqueous environments, such as those based on polylactic acid (PLA) and polyglycolic acid (PGA) [4]. This resorption behaviour offers the potential advantage of the fracture plate disappearing once healing is complete. By combining PLA and PGA as copolymers or blends, and by controlling the concentrations of each component, materials with a range of mechanical properties have been developed, with differences in dissolution times, ranging from months to years [5]. Consequently, fracture plates have been developed that are suitable for use in a number of anatomical areas (e.g., the craniofacial region). However, the inferior mechanical properties of these polymers, in respect to titanium alloys and the bone means that applications for these polymeric fracture plates are limited [4]. The plates often need to be much thicker than Ti-alloy plates designed for similar applications due to the lower stiffness of the polymer plates being insufficient to protect the healing bone without the increased polymer bulk. Additionally, these plates have several (potential) post-operative complications (e.g., acid degradation products can cause inflammation) [6,7,8,9]. Considerable research has been conducted to improve these polymers, to extend the range of clinical applications of these materials and reduce the incidence of post-operative complications.

This review describes the properties of PLA and PGA homopolymers, their copolymers, and blends, together with a review of other polymers that have been added to improve properties. It also describes the methods used to improve the fracture plate’s mechanical and physical properties, by producing composite materials, which are often made using the addition of glasses and ceramics. Further, we discuss attempts to improve the biocompatibility of these materials by adding bioactive components or functionalisation of already existing components of the polymer glass composites.

## 2. Polymer Enhancements

### 2.1. Copolymers and Polymer Blends

Copolymers are favourable as they combine the beneficial properties of the monomeric species whilst simultaneously negating their disadvantageous properties. The same applies for polymer blends and the polymers that constitute these blends. This results in the possibility of a range of materials with desirable properties, including tailored material degradation rate, tunable biophysical, and biochemical properties [10]. Over the years, there has been a wide range of copolymers investigated for bone fixation applications, with copolymerisation being the key feature. Some of the most common polymers investigated include poly-l-lactide (PLLA), poly(d-lactic acid) (PDLA), poly(d,l-lactic acid) (PDLLA), and polyglycolic acid (PGA). These are summarised in Table 1.

**Figure 1:**
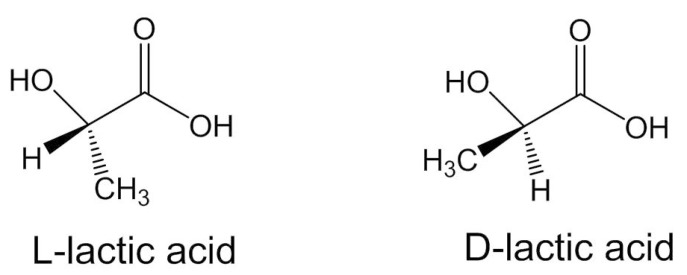
Enantiomer structures of PLA monomer [32].

Polylactic acid (PLA) is an aliphatic, biodegradable polyester used in a range of biomedical applications [21,22,23]. PLA polymers are used in bone fixation applications as it is possible to tailor the rate and degree of crystallinity, and, thereby, mechanical properties, degradation behaviour, and processing temperatures [5]. These are achieved through controlling the stereochemical architecture and the molecular weight of the polymer. Two stereoisomers of PLA are PLLA (poly(l-lactide) and PDLA poly(d-lactide) (Figure 1). PDLA is amorphous because of the racemic mixture of monomer, which disturbs crystallinity; consequently, erosion occurs at a significantly faster rate compared with PLLA [24,25,26,27]. To compare the two, a highly crystalline PLLA will take 2–5 years [28] to degrade in a normal physiological environment in vivo and in vitro (pH 7.4, phosphate-buffered solution (PBS), and incubated at 37 °C) [29]. An amorphous poly(d,l-lactic acid) (PDLLA) shows loss of integrity in 2 months and complete degradation in 12 months under similar conditions [30,31].

PLLA and PDLA have been used extensively as copolymers to create materials for bone fixation applications [20,22,23,33,34]. They can also be combined to form poly(d,l-lactic acid) PDLLA, with ratios of each polymer being varied. By controlling L/D ratios, a variety of different mechanical properties and degradation profiles can be obtained and implemented for bone fixation at various sites [35]. Poly-l/dl-lactide (70/30) plates have been successfully used in maxillofacial applications where an enhanced degradation rate is necessary to eliminate oedema formation; which can occur as a result of the slow degrading PLLA [11]. The strength of these plates is comparable to those of PLLA. Poly-D (2%),L (98%)-lactide interference fixation screws have been used in clinical applications, displaying no adverse side effects and a good rate of degradation due to the D isomer in the copolymer [12]. Superior thermal and mechanical properties, compared to 100% pure l- or d- lactide polymers, have been reported from the production of stereo-complexes of enantiomeric PLAs. A polymer stereocomplex can be defined as “a stereoselective interaction between two complementing stereoregular polymers, that interlock and form a new composite” [36]. This results in a material with enhanced physical properties due in part to the formation of crystallites with intermolecular crosslinks [5,26,37,38,39].

Self-reinforcement (SR) is a form of copolymerisation where a composite structure is formed from a polymer matrix being reinforced with oriented reinforcing units, such as fibres, fibrils, or extended chain crystals. These units are composed of the same material as the matrix [40]. This technique can produce a range of polymeric devices of very high strength, including poly-l/dl-Lactide (SR-P(L/DL)LA) 70/30, which was developed for use as miniplates and miniscrews for anterior mandibular fracture fixation [40]. The matrix material, PLDLLA, was formed into a composite structure by reinforcing with the same material (PLDLLA) and was reported to have bending strengths of up to 400 MPa [40]. Self-reinforced materials were developed and enhanced through controlling copolymer ratios (L/D). Through blending the L/D isomers, it is possible to obtain fracture fixation plates that are bendable at room temperature without the application of heat, which can be required for other types of non-ductile resorbable materials used in fracture fixation [22,41,42,43]. SR-PDLLA/PLLA (40 PDLLA:60 PLLA) showed faster absorption in vivo compared with SR-PLLA, possesses sufficient mechanical properties for fixation of osteotomies, and displays no adverse foreign body reactions at 48 weeks post implantation [44]. Highly oriented polyester fibres were used as reinforcement in self-reinforced polymers or single polymer composites [45]. PLLA matrices with PLLA fibre reinforcement produced materials with increased strength values compared to unreinforced PLLA [46,47] (bending strength of 200 MPa and shear strength of 94–98 MPa), appropriate for treatment of the cortical bone in certain fixation sites [48]. Self-reinforced P(L/DL)LA plates and screws of 70:30 P(L/DL)LA (BioSorb Fx, BionX Ltd., Bedford, MA, USA) were clinically assessed for bone fixation with minimal post-operative complications [49] in craniomaxillofacial [40] and limb surgery [50]. Self-reinforced PLA was successfully used as fixation plates and screws in the treatment of children’s forearm fractures, demonstrating stability in a long arm cast, and supporting osteosynthesis whilst eliminating the need for a second surgery [51].

Polymer blends involve the physical entanglement of at least two polymers in the mix. PLLA has also commonly been combined with PGA for the purpose of bone fixation applications. PGA is a rigid material with high crystallinity, which translates to a high strength and modulus, but also has a faster degradation rate than PLA, on the scale of 6–12 months [52]. When combined with PLLA, a desirable blend with good mechanical properties is formed. PLLA/PGA (82:18) has been used in widespread craniofacial applications due to its ability to maintain sufficient mechanical strength for 6–8 weeks, with in vitro studies showing mass loss being complete in 9–15 months [13]. When PGA is combined with PLA, the copolymer has a balance of the hydrophilic properties of PGA and PLLA, leading to an intermediate rate of hydrolysis. Higher incorporation of PGA may elicit a faster degradation rate, but this is not always the case. One study found that a content of PLA between 75 and 100% and PGA 0–25% was the most desirable in terms of degradation characteristics [14]. Clinically, LactoSorb copolymer pins, composed of PLLA/PGA (82/18) are used in an array of orthopaedic applications [13]. This material has a strength loss profile that better mimics the healing process in comparison the homopolymer PGA or PLLA. A degradation rate slower than that of PGA reduces the occurrence of premature fixation strength loss before bony union occurs, whilst a faster degradation rate than PLLA allows for a faster transfer of load to the surrounding healing tissue, promoting remodelling. The PLLA/PGA pins were used successfully in distal chevron osteotomies, providing fixation on a level comparable with metallic implants [53].

Blending lactide polymers with non-lactide polymers were investigated in order to alleviate the brittle nature of the former materials. Poly-l-lactide/ε-caprolactone (PLCL), trimethylene carbonate (TMC), and poly(3-hydroxybutyrate-co-3-hydroxyvalerate) (PHBV) have all shown promise in reducing the brittle character of PLLA specifically. Poly dl-lactide/glycolide copolymer (PDLGA) and poly-l-lactide/ε-caprolactone (PLCL) were combined to form a set of PDLGA/PLCL blends. Greater incorporation of PLCL into the blend provided a decline in elastic modulus and yield stress and an increase in yield strain. Characteristics were provided from the elastic polymer, PLCL. When PLCL makes up 60% of the polymer blend, the brittle nature of the polymer is eradicated [19]. A novel terpolymer based on l-lactide, d,l-lactide, and trimethylene carbonate (TMC) displays brilliant mechanical and biocompatibility properties of PLDLA. TMC, an elastomeric component, was incorporated in with the lactide polymers to alleviate the brittle nature. The three components were first copolymerised and then moulded into pins for evaluation. Incorporation of TMC into the blend reduced mechanical properties of the material however make it suitable for use in soft tissue applications [17]. PDLLA was combined with copolymer of trimethylene carbonate (TMC) and e-caprolactone (CL) to form a PDLLA/P(TMC-CL) blend, assessing its suitability for fracture fixation by studying degradation. The material, manipulated into bone plates and screws, was tested in vitro and in vivo (mandibular fractures). In vitro, the initial tensile strength was maintained for a time-period after bone healing occurred at 6–12 weeks. At 45 weeks of the study, it was seen that minimal water was absorbed by the degrading blend, with no observation of significant mass loss. In vivo, the implants devised enabled uneventful bone healing and no premature failure of the devices were seen; however, although undisturbed bone healing occurred around 12 weeks, the implanted screws had broken and bone plates loosened [20]. Inion (Finland) at present has clinically available products on the market that are composed of l-Lactide, d,l-Lactide, polyglycolide, and TMC [54]. Clinical studies using the material in the form of miniplate conclude that the resorbable blend can be used in the same circumstances as titanium miniplate, with exception in maxillary elongation and mandibular setback [55], due to higher relapse in these circumstances and one degradable plate not being stable enough to prevent such an occurrence. Pins composed of PLLA/PHBV were assessed for their mechanical and degradation properties, where the addition of PHBV improved thermal properties and dampened the brittle character of PLLA. Intermediate ratios of the PLLA/PHBV (60/40, 50/50, 40/60) copolymer degraded faster than pure PHBV whilst maintaining mechanical properties for longer than pure PLLA [16].

### 2.2. Orientation

Higher polymer crystallinity and chain orientation can be used to enhance mechanical properties in the direction of orientation [56]. Polymer chain orientation is a way to produce polymers with high strength and modulus. This can be achieved typically through cold- and/or hot-drawing of the polymer materials by melt processing above a polymer glass transition temperature (Tg), but below the melting temperature (Tm) [57].

Processing methods, such as extrusion, compression, and injection moulding can be used to achieve chain orientation. It has been found that processing poly(d-lactide) by solid-state extrusion improved the bending strength and bending modulus of the material. The draw rate and temperature of extrusion are the key parameters that determine the mechanical properties of the polymer. Annealing of the polymers prior to solid state extrusion also allowed for relief of stress concentration and chain orientation [57], and the crystallinity of compression-moulded or extruded PLLA samples were increased through annealing, and sterilisation by ethylene oxide gas processing. Increased crystallinity is associated with an increase in the Young modulus; however, it is also accompanied with a decrease in tensile strength and elongation at break [58]. Optimisation of the injection moulding process and use of nucleating agents can result in an increase of crystalline content of commercial grade PLA from 5 to 42%. An added benefit is the concomitant decrease in processing time [59]. Despite the advantages of processing methods on some of the mechanical properties of material, the molecular weight does diminish with heat treatment of polymers and should be taken into consideration, as this would, in turn, impact the mechanical behaviour of the polymer overall.

Orientation of polymer chains can also be achieved through enhanced entanglement between the long-branched chains. A poly(lactic acid)-branched-poly(lactide-co-caprolactone) (PLA-b-PLCL) was developed through two phase separated structures using long chained branches and demonstrated high tensile strength and modulus (172 MPa and 5.4 GPa respectively) showing potential for use in bone fixation [15].

## 3. Composite Materials

The limited mechanical properties, the lack of bioactive behaviour that allow bone apposition and bonding on the polyester implant, and complications associated with the acidic degradation products are major drawbacks of polyester implants. Ceramic materials are high strength, biocompatible, corrosion resistant, and possess a high level of bioactivity. For this purpose, composite polyester implants with ceramic fillers (hydroxyapatite (HA), tricalcium phosphate (TCP)), silicate, and phosphate glasses have been used to improve the overall mechanical properties and endow bioactivity in bioresorbable osteofixation devices. In addition, alkaline dissolution from bioactive ceramic fillers counteracts the decrease of pH in the ambient implantation site by the acidic degradation products of the polyester matrix.

### 3.1. Particulate Bioceramics

Hydroxyapatite (HA) and β-tricalcium phosphate (TCP), have undergone intensive research for their use in bone applications [60]. HA (Ca_10_(PO_4_)_6_(OH)_2_) has a molar ratio of 1.67 Ca/P and is a bioactive ceramic that is typically used in coatings and can be incorporated into materials in particulate form. HA has been shown to improve the biocompatibility of biomaterials due to its similarity in structure and composition to bone and enamel [61]. It is osteoconductive and osteoinductive [60]. Tricalcium phosphate has three polymorphs; α-TCP, β-TCP, and α′-TCP. In particular, β-TCP displays excellent biocompatibility, bioactivity, and bio-resorbability. Although HA has greater mechanical strength than β-TCP, the latter is more resorbable. This encourages faster growth of new bone around the implant.

Polyesters, such as PLLA, PCL, and PHBV can take up to 2 years to degrade, and ceramic incorporation has been found to decrease this. HA enhances the degradation rate of PCL, with mass loss in vitro increasing with HA content. In addition, the compressive yield strength and modulus of the material also increased almost linearly with HA content [62]. Up to 3 wt% nano-hydroxyapatite (n-HA) incorporated into a poly-l-lactic-co-glycolic acid (PLGA) matrix has been found to significantly increase the mechanical properties of the polymer [63,64]. It was concluded that a higher content of n-HA in the composite promoting enhanced crystallisation, but also caused greater agglomeration in the PLGA matrix, which resulted in a decrease in mechanical properties. The addition of 3 wt% n-HA enhanced the degradation performance of the material, making the material promising for use in clinical applications in comparison to pure PLGA in bone fracture internal fixation materials. Porous polymer-hydroxyapatite scaffolds for femur fracture treatment produced by 3D printing have presented promising results. Hydroxyapatite nanoparticles were used to enhance PLA polymer and subsequently 3D-printed into porous cylindrical structures. Comparing 5% to 25% HA content in PLA, the compressive modulus and elastic modulus increased by ~38% and ~92%, respectively, a 11% decrease in porosity of the scaffolds was also seen. These results illustrate the reinforcing behaviour of HA and the mechanical properties being a function of porosity [65]. The mechanical and thermal properties of injection-moulded poly(3-hydroxybutyrate-co-3hydroxyhexanoate) (P(3HB-co-3HHx))/hydroxyapatite nanoparticles (n-HA) parts for use in bone tissue engineering and bone fixation were assessed [66]. The addition of 20% nHA improved the mechanical properties; specifically, the tensile moduli and flexural moduli by approximately 60%. A larger content of n-HA leads to agglomeration, with the ductility, toughness, and thermal stability of the material declining. The addition of nHA up to 10% most closely resembled natural bone in terms of strength and ductility and demonstrated controlled agglomeration. Considering its biocompatibility, the copolymer composite can be appropriate as a bioresorbable implant for low-stress bone fixation sites. An alternative way to simple melt mixing of ceramics with polyesters is covalently linking the two. A novel study surface first grafted nano-HA to PLLA. This nano-HA/PLLA composite was then blended with PLLA. Surface-grafting HA to PLLA led to an improvement in mechanical properties, compared to similar non-grafted PLLA/HA composites due to the grafting providing strong linkages between the HA particles and the PLLA matrix [67]. Further works by the researchers saw surface modification of carbonate hydroxyapatite particles with PLGA, with processing as previously described [68]. The PLGA/g-CHAP nanocomposites displayed improved mechanical properties in comparison to unmodified PLGA. This was attributed to the strong interfacial bonding between PLGA and g-CHAP particles. At 2% of g-CHAP content, the fracture strain was increased from ~5% (for neat PLGA) to 20%. When g-CHAP content was between 2 and 15%, the composites showed enhanced tensile strength and fracture strain. The tensile strength decreased linearly with filler content beyond 20%.

In vitro, TCP incorporation into PLLA showed good biocompatibility and a rate of degradation consistent with bone healing [69]. In particular, material strength was maintained for 16 weeks, after which a decline was observed with no measurable strength of the material at 40 weeks. There was a decrease in tensile strength of the material over the investigated period. In vivo, these results were seen on a shorter time scale. The addition of 10% volume TCP filler to PLLA gave a biocomposite material with an extended strength and molecular weight retention period, both in vitro and in vivo [69]. The same experiments conducted on PHBV showed slower degradation rates, which was attributed to the hydrophobic nature of PHBV. Ternary blends of PCL/PGA/tricalcium phosphate (TCP) (80/10/10 and 70/10/20) displayed success for use in low load bearing applications, such as maxillofacial surgery [70]. Adhesion strength of the materials was tested using a previously established protocol, where investigated biomaterials were melted and applied to two bone sections before curing. The mechanical properties of the set fixation was then tested through compressive and tensile force [71]. The blends retain the adhesive strength of PCL whist having improved hydrophilicity. A higher incorporation of TCP also results in enhanced degradation and support for osteoblast growth. PLLA was strengthened with a poly(ε-caprolactone-co-l-lactide) copolyester [18]. A higher quantity of copolyester (PCL/LLA) in the blend provided a greater elongation at break with a concurrent decrease in the Young’s modulus and strength. Use of a chain extender (Joncryl^®^ ADR 4368, BASF, Thailand) enhanced phase compatibility of PLLA and the interspersed copolyester phase.

### 3.2. Glass Fibres

Bioactive glasses were first developed in 1969 and represent a group of materials that have the capacity to bond with bone in physiological environments. They have been greatly studied due to their desirable characteristics, which include biocompatibility, degradation, and mechanical strength [72]. By changing the composition of glass, it is possible to obtain a range of mechanical properties and high controllability over the resorption and ion dissolution that resemble and complement the mineral content of bone. For instance, it is possible to decrease degradation rates in a phosphate glass composition by addition of hydration-resistant metal oxides, such as Al_2_O_3_, Fe_2_O_3_, and TiO_2_ [73,74]. The use of high aspect ratio fibrous bioactive glass structures, as reinforcing phases, has produced fully bioresorbable polyester composites with higher mechanical properties compared to composites with particulate reinforcement [74]; however, studies have shown variable results, with some showing a rapid loss of mechanical properties with degradation [75]. The use of fibres in bioresorbable composites has also been associated with an immediate osteoinductive effect owed to the presence of the filler in the outer surface of the polyester implant [76]. Glass fibre reinforced polyester composites have also been found to act protectively to the polyester matrix against gamma radiation from deterioration of its mechanical properties [74].

The properties of the composite for a given polyester matrix and glass fibre reinforcement depend on the geometry (i.e., aspect ratio) of the filler, distribution within the matrix, volume fraction, and surface area of the composite and strength of the fibre–matrix interface. Therefore, fibre and composite manufacturing methods significantly influence the properties of the composites. Glass fibres are typically fabricated into continuous fibres via melt spinning or preform drawing manufacturing processes, with single filament production being more common [77]. The mechanical, thermal, and degradation properties of glass fibres vary from those of bulk glasses depending on processing temperatures, drawing speed and ratio, and viscosity of molten glass, which ultimately determine the fibre diameter. A smaller fibre diameter leads to increased dissolution times, a fact that is associated with the active surface area of the filler [74].

The fibres can be appropriately aligned within the polyester matrix to create long-fibre composites, or chopped and dispersed within the matrix to create short fibre composites. The use of long fibre reinforcement is associated with improved mechanical properties in the composites when measured along the axis of the fibre. During manufacturing, the fibrous preform has to be positioned within a mould cavity and infiltrated with polyester matrix, which might require complex tooling. Short fibre composites are easier to process with extrusion/injection or casting processes, with the filler is melt-mixed or dissolved with the polyester matrix prior to processing. Solvent casting methods have also been used to create short glass fibre polyester composites. The method of incorporation of glass fibres into the polyester matrix plays an important role in the obtained mechanical and degradation properties.

Long-fibre unidirectional (UD) woven mats and randomly orientated short fibre non-woven mats (RM) of iron doped glass phosphate fibres as reinforcements in a PLA matrix have produced bioresorbable composites with enhanced mechanical properties [78]. UD matt reinforced composites with filler volume fraction of 20% revealed a faster depletion of mechanical properties during degradation compared to the randomly oriented short fibre matt composites of 30–40% volume fraction. A maximum modulus and strength for the RM and UD were 10 GPa/120 MPa and 11.5 GPa/130 MPa, respectively, falling short of the ideal properties for cortical bone. Increased concentration of fibres, despite allowing for enhancement of mechanical properties of the composite material in comparison to polymer alone, also lead to an increased degradation time. Further work demonstrated that ~30% fibre volume fraction of unidirectionally and randomly aligned fibres into PLA rods imparted the composite with a higher initial modulus, which succumbed to degradation faster than PLA alone [79]. The RM and UD-filled PLLA was manufactured into intramedullary nails and the mechanical properties were assessed [80]. The composite reinforced with unidirectionally aligned fibres provided enhanced mechanical properties compared to pure PLA rod. The method of processing via forging at 100 °C also improved the properties of the PLA matrices by influencing chain orientation. This method has a similar effect to the drawing process at low draw ratios. Fibre incorporation and material processing were jointly responsible for the property enhancement. The materials were also processed into bioresorbable screws with promising results [81,82].

Different treatment processes performed on phosphate glass fibres further impact their performance in composites. When short glass fibres were incorporated into PLA in the form of randomly oriented non-woven mats, there was a mass loss of 14% and 10%, respectively, for non-treated and heat-treated fibre composites over 6 weeks, in comparison to no mass loss seen for pure PLA. Incorporation of glass fibres enhanced the material modulus (2.5 GPa → ~ 5 GPa for both composites) and flexural strength significantly, with the latter matching that for cortical bone. Concerning the retention of mechanical properties over 6 weeks of in vitro degradation, the strength of PLA declined slightly, while the modulus was maintained. For the non-treated and treated samples, there was a significant decline in both modulus and flexural strength; 0.5–1 GPa and ~40 MPa, respectively. There was no mass loss for PLA over the 6 weeks compared with ~12% and ~14% for heat-treated and non-treated, respectively. Heat treatment of the fibres led to a decreased dissolution rate. The PLA alone and heat-treated composites displayed higher cell viability due to their slower degradation [83]. Incorporation of short non-treated and heat-treated glass fibres in the form of randomly oriented non-woven mats was also investigated in poly-caprolactone. The composite materials presented with a flexural strength and modulus of up to 30 MPa and 2.5 GPa, respectively, values that are comparable to those of the human trabecular bone. A higher mass loss was seen in the composites with a higher volume fraction (V_f_ 17/18%), 20% compared with 8% for (V_f_ 6.4%) over 5 weeks [84]. The rapid decrease in mechanical properties in glass fibre reinforced polyesters is attributed mainly to early hydration of the reinforcement due to weak interfacial interactions and polymer swelling during degradation that increases the internal stress of the system inducing early cracking and failure [85,86]. Coupling agents, including silanes, acids, and other agents that can create covalent bonds between filler and matrix material have been used to enhance the interfacial properties between phosphate glass and PLA matrices. The improved interfacial shear strength between the phosphate glasses and PLA matrix prevents early hydration of the filler and assists with the fibre/matrix load transfer, thereby improving the overall mechanical properties of the composite material [87].

Two main points can be seen from assessing composite material. Firstly, the method of incorporation of bioceramic fillers into the polymer matrices must be considered, as studies have shown certain processes can be disadvantageous to mechanical properties due to improper blending and homogeneity across the composite material [88]. Secondly, the volume fraction of the filler must be optimised, as excessive content can lead to agglomeration and depletion of mechanical properties.

## 4. Surface Enhancement

### 4.1. Overview of Surface Enhancements

Surface modifications for bone fixation has been an active area of research for decades and is commonly carried out in order to enhance physiological bone fixation, assist the healing process, and improve biocompatibility, functionality, and biological efficacy. The success or failure of the implant is dependent on the device and surrounding tissue at the implant interface [89]. Fabrication processes can change the surface composition in comparison to the material bulk. This may be due to oxidation or hydrolysis of surface groups and/or preferred molecular orientation of surface groups in order to minimise surface free energy. Such effects typically occur unevenly over a surface, which can impact the performance of the material [90]. It is thereby crucial to alter polymer surfaces to be able to regulate concurrent surface interactions and responses.

Controlling biocompatibility is an ongoing challenge with biomaterials, as synthetic and naturally occurring polymers quite often do not have the surface properties, which are required for specific applications [91]. Generally, surface enhancements are made with certain objectives. When considering polyester surface enhancements, they are challenging to modify due to their ease of degradation with chain scission, solvent sensitivity, and low heat stability [92,93]. Surface enhancements are typically conducted to increase or reduce [90]:

- Hydrophilicity;
- ionic charge/pH;
- adhesion of microorganisms;
- adsorption of molecules;
- permeation of molecules;
- roughness;
- impurities;
- chemical/ biological reaction kinetics.

Surface engineering generally includes alteration of topographical (i.e., roughness) and chemical (i.e., coating) characteristics of a medical device. An increased roughness is desirable as it leads to an increased surface area, which in turn gives a larger area for cell adhesion. It additionally enhances biomechanical interlocking between bone tissue and implant [89]. Calcium phosphates are typically the group of materials used for coating orthopaedic implants due to the excellent bioactivity of these ceramics. Together with the mechanical advantages of the substrate implant, there is an improvement of the implants overall performance [94].

### 4.2. Surface Enhancements of Polyesters for Bone Fixation

**Figure 2:**
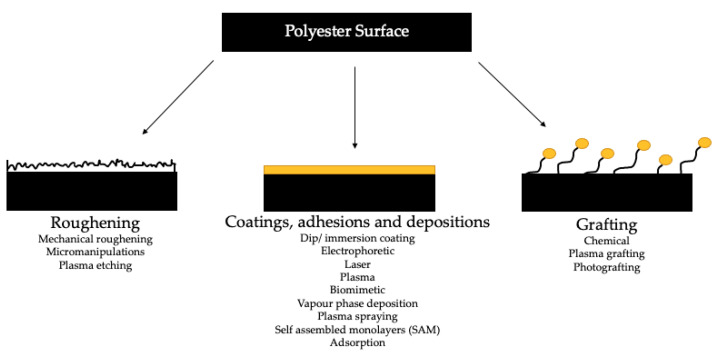
Polymer surface modification methods (for biodegradable polymers) [89,91,98,99,100,101].

From reviewing literature and the commercial products, which are available on the market for bone fixation (Table 2), it is apparent that surface enhancements of polyesters used in bone fixation are not yet employed. A vast array of in vivo and in vitro work has been conducted on bone tissue engineering applications, but this has not yet been thoroughly investigated in bone fixation materials and devices. Figure 2 illustrates the array of techniques that can be used to alter surface properties. These techniques have been divided into three categories: (i) roughening, (ii) coatings, adhesions and depositions, and (iii) grafting. Each group of techniques allows for different topographical surface enhancements. It can be difficult to assess which technique is the best fit, for the purpose of enhancement, for a specific application. Work conducted on plasma, chemical, or laser methodologies investigated what treatment was most appropriate for the modification of PLA surfaces. The effects of each treatment was looked into mechanically (with surface roughness analysis), surface wettability, and chemically (via XPS). Chemical treatment caused the most drastic increase in surface roughness. Plasma treatment lead to an increase in roughness and was found to be dependent upon exposure time. Laser treatment appeared to decrease surface roughness when compared to the untreated PLA. Given these results, chemical modification may be an appropriate method to be used on PLA joint implant surfaces, as an increased surface roughness leads to an increased strength of the joints. Materials processed with plasma exhibit an oligomeric layer on the surface, which could be detrimental to the adhesive joint formation process. As laser-treated surfaces showed a decrease in surface roughness, this method was not deemed suitable to use with regards to material mechanical properties. Chemical modifications cause the least change in water contact angle, but plasma and laser treatments show a significant increase. Plasma treatment overall induces the higher surface energy of the three treatments. Oxygen content of the surfaces increases with all three modes, with plasma processing giving the highest. Plasma modifications are deemed to be the most beneficial of the three processes for polymer implants [95]. Plasma modifications are known to be an effective method to treat the surface of polymers for biomedical applications as these treatments can be selective, yet not affect the bulk polymer characteristics [96,97]. As shown in Figure 2, plasma can be used to introduce surface roughness, graft surfaces, and deposit material onto a surface. Plasma surface modifications often offer a shorter processing time in comparison to other surface modification methods. Although both plasma treatment and plasma coating technologies are commercially available for modifying polymeric surface properties, their use in fracture fixation applications has not been significant to date. 

There is a large scope of data regarding the biocompatibility of polyester materials, specifically with regards to surface enhancement [101,102,103,104,105]. As the polymer biomaterials have interaction directly with an extracellular environment, it is vital that an adverse immune response is not provoked [91,100]. A few important surface characteristics can be altered to obtain the right biocompatibility control for the intended application. These include surface morphology, chemical structure and functional groups, interfacial free energy, wettability, cytotoxicity, and adhesion [91,105]. Plasma treated PDLLA was combined with collagen anchorage. The plasma surface property alterations included improved surface hydrophilicity and increased surface energy. As a result of plasma pre-treatment, there was more collagen fixated to the surface. Mouse fibroblast cells were used to assess cell affinity and showed good affinity with the plasma treated, collagen anchored samples in comparison to untreated surfaces [106]. Similar enhanced cell affinity behaviour on PDLLA has also been reported after treatment with anhydrous ammonia plasma treatment [107]. In contrast, PLA modified with medium pressure dielectric barrier discharge (DBD) plasma treatment illustrated an increase in hydrophilicity along with an increase in oxygen content, measured by contact angle measurements and XPS, respectively. Biologically, plasma modification of PLA led to increased initial cell attachment; however, after 7 days, there was no significant difference in activity on the untreated and treated samples. It was concluded that cell proliferation was not influenced by the application of plasma treatment to the surface [98]. The influence of plasma treatments on these polymer surfaces is dependent on the experiment parameters and materials, which will give a variation in cell attachment success.

A challenge with altering material surfaces is the side effect that may be imposed on the bulk material [91], however; there are surface enhancement techniques that can limit or even avoid this. A method investigated on polyester materials, which may limit the influence on the bulk, is extreme ultraviolet radiation (EUV). Degradation of the bulk material can be limited through using short wavelength radiation, which is in the extreme ultraviolet range; which, in turn, is only absorbed by a very thin layer of the polymer surface (<100 nm) [91]. Vapour phase (VP) grafting of N-vinylpyrrolidone was conducted on four types of biodegradable polymers; PLA, PCL, PLGA, and PTMC. Wettability of all materials was enhanced by the process and it was possible for the surface topographies not to be altered due to the thin graft layer applied. Additionally, film surfaces for the grafted materials with VP after 30 min was rougher than the original polymers and enhanced good cellular adhesion was noted with PLLA, PLGA, and PTMC [99]. Radio frequency (RF) plasma is an additional process for modification without bulk properties being affected [108]. Characteristics, including advantages and disadvantages of some commonly used surface enhancement treatments on polyester surfaces, are summarised in Table 2.

The methods discussed highlight some of the breakthroughs, which have enabled triggering of specific responses, recruitment of the correct cells and stimulus for these cells to perform. These are additional functionalities to otherwise inert polyester biomaterials [89].

## 5. Current Market Products

Table 3 summarises the current polyester-based biomedical devices used in bone fixation applications. It can be seen that the majority of devices are composed of PLA and its composites, which is to be expected, as PLA in clinical applications is the most commonly used biodegradable polymer [111]. This is predominantly due to its excellent biocompatibility, mechanical properties, and history of use in medical applications since the 1970s [112]. The majority of products that are detailed are used in small load bearing applications, which suggests that material enhancements to biodegradable polymers are still a requirement in order to be used in larger load applications. This may in part be due to the biodegradability and mechanical properties, prior to and during degradation, of these materials, when scaled into larger devices.

## 6. Final Considerations and Perspective

Table 4 summarises the enhancement strategies, material/s, and the resultant modified properties, as discussed in this review.

The main reasons for seeking to enhance the properties of materials for fixation are:

- Modifying the modulus, to allow either the stiffness of the device to be changed, or to allow stiffness to be maintained whilst using less material.
- Modifying the strength, again to change the strength of the device or to allow the strength to be maintained whilst using less material.
- Making the material less brittle, to avoid brittle failure in use or when fitting the device.
- Changing the degradation rate of the material to alter the time for a device to resorb.
- Changing the degradation chemistry to avoid an excess of acidic degradation products.
- Improving the interaction between the device and native tissue.

For the most part, enhancements have been applied using PLA or PLLA as a comparator material, aiming to retain strength, reduce brittleness, increase degradation to promote resorption, mitigate the acidic degradation products, and promote better integration between the polymer and the native tissue.

The complexity arises from the inter-related nature of the properties, which are being manipulated: most of the enhancement methods previously described affect more than one of the properties of interest, and the device design can be used to mitigate some effects. For example, most devices are relatively thin, which enhances degradation and limits the volume of degradation products released at the implantation site.

From Table 2 it is clear that two key material enhancement strategies have been adopted broadly adopted clinically, and so can be seen to offer benefits with clinical value:

- Copolymerisation and blending of polymers for greater flexibility and increased degradation rates, with use of glycolic acid and variations in the use of l- and d-lactic monomers or polymers, the most common approaches in clinically applied devices.
- Composite materials with particulate bioceramics offer benefits in terms of mitigating acidic degradation, and increasing degradation rate, whilst having a relatively small effect on the other properties, although care must be taken to ensure that the amount of particulate loading is not so high as to make the material brittle.

The use of materials with anisotropic mechanical properties, through orientation of the polymer or through long fibre reinforcement, has not been adopted clinically, perhaps suggesting that the loading that the devices are put under in practice is too complex to be addressed by enhancement in one orientation.

In terms of future developments, there is ongoing interest in blending new polymers with polyhydroxyalkanoate-based materials, popular for their green production route, biocompatibility, and benign degradation products [113]. There is also ongoing interest in refinements to particulate bioceramic additives, in terms of the additive itself, with Mg and Sr substituted HA systems explored, and in terms of the form of the reinforcement, with both nanoscale additives and short fibres being explored for enhanced biocompatibility and degradation profiles.

Surprisingly, surface modification techniques have not been significantly explored for fixation devices, despite the more general growth of interest in using surface functionalisation methods to enhance medical devices [114]. Reported work to date has predominantly focussed on promoting hydrophilicity on surfaces, but it is possible to apply specific molecules onto surfaces to elicit specific responses in vivo. The reasons for the application may be to generate a more biomimetic surface for attachment to the native tissue, or to release stimulatory or therapeutic molecules to the fracture site, and exploration of the potential for this in fixation applications could offer a way to further improve the performance of this important class of medical devices.

## Figures and Tables

**Table 1:** Summary of Polymer Blends.

Polymer	Properties	Study Type	Degradation	Clinical Application	Reference
Poly-l/dl-Lactide [(P[L/DL]LA)] (70/30) Copolymer	Sufficient to support fractures, bendable	In vivo, human	Consistent with bone healing	Orbital fractures	[11]
P(D(2%),L(98%))lactide Copolymer	Sufficient to support fractures, bendable	In vivo, human	Consistent with bone healing	Interference fixation screws for anterior cruciate ligament surgery	[12]
PLGA (l-lactide 82: glycolide 18) Copolymer	7 GPa Young’s modulus 50% by 12 weeks	In vitro	50% decline in mechanical properties by 12 weeks, peak retention at 8 weeks	Choice of material in foot surgery	[13]
PLGA/PLA (100:0, 75:25, 50:50, 25:75, 0:100) Copolymer	N/A	In vivo, rodent	2 weeks–6 months	Oral resorbable implants	[14]
Poly(lactic acid)- b-poly(lactide- co-caprolactone) (PLA-b-PLCL) 30 wt% PLCL Copolymer	173 MPa tensile strength 5.4 GPa Young’s modulus)	In vitro	N/A	Smart bone fixation material with shape memory effect	[15]
PLLA/PHBV (40:60) Blend	Improved elasticity compared to PLLA	In vitro	PLLA: 12 weeks, PHBV: 53 weeks	Orthopaedics	[16]
P(L/D,L)lactide/TMC (56:24:20 and 49:21:30) Copolymer	Decrease in Young’s modulus and tensile strength compared to P(L/D,L)LA (0.9 GPa from 3.1, 27 MPa from 50 MPa)	In vitro	N/A	Soft tissue engineering	[17]
Poly-e-caprolactone-co-l-lactide (100:0, 90:10, 80:20, 60:40) compatibilised with 2.0 phr Joncryl^®^ Blend	Young’s modulus/stress at break: 100:0—1.5 GPa/57.6 MPa 90:10—1.2 GPa/44.8 MPa 80:20—1.1 GPa/41.8 MPa 60:40—0.32 GPa/14.6 MPa	In vitro	N/A	Long term implantable devices, tissue engineering, drug delivery	[18]
Poly(d,l-lactide-co-glycolide)/(l-lactide-co-ε-caprolactone) (PDLGA/PLCL) PDLGA(dl-lactide/glycolide, 53/47 M ratio), 70/30 l-lactide/ɛ-caprolactone M ratio PDLGA/PLCL (80:20, 60:40, 40:60, 20:80) Blend	Young’s modulus/Yield strength PDLGA—1.2 GPa/36 MPa PDLGA:PLCL(80:20)—1.1 GPa/28 MPa PDLGA:PLCL (60:40)—0.6 GPa/19 MPa PDLGA:PLCL (40:60)—0.02 GPa/5.6 MPa PDLGA:PLCL (20:80)—7.1 MPa/-	In vitro	Degradation accelerated by larger amounts of PLDGA. PDLGA has a lower molecular weight compared with PLCL; therefore, favours an increased hydrolytic degradation rate	Minimally invasive surgery, shape memory polymer	[19]
PDLLA/P(TMC-CL) (Poly(l/d-lactide) (85:15)/20% wt (50/50 trimethylene carbonate-co-e-caprolactone) Blend	Decrease in tensile strength (50 MPa (PLDLA) in comparison to 30 MPa (PLDLA20%P(TMC)CL)), bending modulus increase (2.7 GPa to 4.9 GPa), elongation increase (7.5% to 130%), increase in impact strength	In vitro and in vivo, canine	No significant mass loss up to 45 weeks in vitro, in vivo healing within 12 weeks, screws and plates loosened after 18 weeks	Single fractures of the mandible	[20]

**Table 2:** Summary of surface enhancement treatments on polymers [89,102,105,109,110].

Process	Advantages	Disadvantages
Chemical grafting	Exposure of functional groups on material surface. Long and stable effects produced.	Limited by the functional groups on the surface. Chemical modifications (i.e., aminolysis/hydrolysis) may be required prior to fixation of biomolecules, causing destruction of the topological structure of the surface.
Pulsed Laser Deposition (PLD)	Simple, versatile, rapid, cost effective. Precise control of the thickness and morphology of the films deposited.	Small area of structural and thickness uniformity.
Photografting	Solvent free approach. Non-destructive Surface topography maintains a thin graft layer.	May affect the material bulk properties and induce material degradation.
Extreme UV radiation (EUV)	Penetration depth limited (<100 nm in upper layer of polymers), affect surface layers only. Strong interaction of EUV photons with material.	Lack of commercially available lab sources of EUV radiation. High equipment costs.
Plasma Modification	Simple and widely used. Bulk properties are not affected.	Size of treated material restricted by the size of the treatment chamber.
Physical coating	Simple and effective methodology.	Bonding relatively weak, specifically in aqueous environments.

**Table 3:** Orthopaedic fracture fixation devices on the market.

Company	Device	Application	Material
CONMED	SmartPin/SmartPin PDX	Foot and ankle.	PLA
	SmartNail	Foot and ankle.	PLA
	BioScrew	Knee (tibial/femoral applications). Bioabsorbable interference screw.	PLA
	BioMini-Revo	Shoulder.	PLA
J&J	RAPIDSORB	Resorbable plates, meshes and screws intended for use in fracture repair, and reconstructive procedure of the craniofacial skeleton. Implants resorbed in 12 m.	85:15 poly(l-lactide-co-glycolide)
	ORTHOMESH	Resorbable graft containment system.	85:15 poly(l-lactide-co-glycolide)
Stryker	SonicPin	Austin/chevron osteotomy. Maintain alignment and fixation of bone fractures, osteotomy, or bone grafts in hallux valgus applications in the presence of appropriate immobilisation (e.g. rigid fixation implants, cast and brace.)	PLDLLA
	Delta System	8–13 months, craniofacial and mid-facial skeleton fixation.	P-L/D-LA/GA
Smith & Nephew	Regenesorb	Material used in orthopaedic applications.	PLGA with calcium sulphate and β-TCP
	SureTac III system	Shoulder.	
Teijin Medical Technologies	OSTEOTRANS-OT	Orthopaedic and thoracic surgery. Products include screw, pin, washer, interference screw, rib/sternum pin.	µ-HA and PLLA
	OSTEOTRANS-MX	Products include meshes and screws. Used in cranio, oral, and maxillofacial, plastic and reconstructive surgeries.	µ-HA and PLLA
	FIXSORB	Used in cranial, oral, maxillofacial, plastic, and reconstructive surgeries. Products include screws, washers, pins, rods.	PLLA
	FIXSORB MX	Used in cranial, oral, maxillofacial, plastic, and reconstructive surgeries. Products include plates and screws.	PLLA
Gunze (Japan)	GRAND FIX	Oral, craniomaxillofacial, and plastic surgery. Products include plates and screws (mini for plate locking and cortical full thread screws), rib pins (rib and sternum fixation) and pins, screws, and ACL screws.	PLLA
Acumed (USA)	Biotrak Screws	Fixation for small bones and bone fragments in the upper and lower extremities, including fractures, fusions, and osteotomies. Composed of Biotrak helical nail, pin, standard, and mini screw.	PLLA
Arthrex (USA)	Trim it spin Pin	Pins. Foot and ankle.	PLLA
Takiron	Fixsorb	Fracture fixation,	PLLA/HA
Biomet Arthrotek	Bio-Phase Reunite Screws, pins plates	Fracture fixation,	PLLA/PLG
	LactoSorb		PLLA/PGA

**Table 4:** Polyester enhancement strategies for bone fixation bioresorbable polyester materials.

Enhancement Strategy	Enhanced Polyester Material	Reference	Modified Properties
Self-reinforcement (oriented units)	SR-P(L/DL)LA	[40,49,50,51]	Strength and elasticity, thermal properties, degradation/absorption profile, retention of mechanical integrity
	SR-PLLA, SR-PDLLA/PLLA	[22,44]	
	SR-PLA	[46,47,48]	
Copolymers/blends	PLA stereocomplexation	[37,38,39]	
	P(L/DL)LA (70:30)	[11]	
	PDLA (2:98)	[12]	
	PLLA/PGA	[13]	
	PLA-b-PLCL	[15]	
	PLLA/PHBV	[16]	
	P(L,DL)LA/TMC	[17]	
	PLLA/(PCL/LLA)	[18]	
	PLDGA/PLCL	[19]	
	PDLLA/P(TMC-CL)	[20]	
Tuning of thermoforming parameters, nucleating agents, thermal post-processing	PDLA	[57]	
	PLLA	[7,58]	Chain orientation and crystallinity
Bioceramic reinforced co/polyester composites	PLLA/TCP, PHBV/TCP	[69]	Strength and elasticity, thermal properties, degradation/absorption profile, retention of mechanical integrity, endowment of bioactivity (osteoinduction)
	PCL/HA	[62]	
	PLGA/nHA	[63]	
	PLLA/PLLA-grafted HA	[67]	
	PLGA/PLGA grafted CHA	[68]	
	P(3HB-co-3HHx)/nHA	[66]	
	PLGA/nHA, TCP, Mg-CP, Sr-CP	[88]	
	PLLA/phosphate glass fibres	[78,79,80,81,82,83]	
	PCL/CP glass fibres	[84]	
	PLA/CP (coupling agents)	[87]	
Surface functionalisation	Covalent grafting techniques on PLLA, PCL, PLGA, PTMC substrates	[96]	Surface topography and roughness, surface free energy, and chemistry to improve cell adhesion and proliferation and/or induce specific responses
	Chemical/plasma/laser on PLA substrates	[95,99]	

## Abbreviations

Polylactic acid	PLA
Poly (l-lactic acid)m	PLLA
Poly (l,d lactic acid)	PLDLA
Poly (d-lactic acid)	PDLA
Poly (d,l-lactic acid)	PDLLA
Poly(l, dl-lactic acid)	PLDLLA
Poly(glycolic acid)	PGA
Poly-l-lactic-co-glycolic acid	PLGA
Poly(lactic acid)- b-poly(lactide- co-caprolactone)	PLA-b-PLCL
Poly(3-Hydroxybutyrate-Co-Hydroxyvalerate)	PHBV
Tri-calcium phosphate	TCP
Calcium phosphate	CP
nano/carbonated/hydroxyapatite	n/C/HA
Poly(trimethylene carbonate)	PTMC
